# Association Between Physical Tests and Patients-Reported Outcomes in Athletes Performing Exercise Therapy for Patellar Tendinopathy: A Secondary Analysis of the JUMPER Study

**DOI:** 10.1177/03635465231200241

**Published:** 2023-10-10

**Authors:** Jie Deng, Stephan J Breda, Denise Eygendaal, Edwin HG Oei, Robert-Jan de Vos

**Affiliations:** † Department of Orthopedics and Sports Medicine, Erasmus MC University Medical Center, Rotterdam, the Netherlands; ‡ Department of Radiology and Nuclear Medicine, Erasmus MC University Medical Center, Rotterdam, the Netherlands; Investigation performed at Erasmus Medical Center, Rotterdam, the Netherlands

**Keywords:** overuse injury, exercise rehabilitation, physical functional performance, VISA-P, prognosis

## Abstract

**Background:**

Physical tests are commonly used in patellar tendinopathy to aid the clinical diagnosis, assess the prognosis, and monitor treatment. However, it is still unknown whether these physical measures are associated with patient-reported outcomes after exercise therapy.

**Purpose:**

To identify the prognostic value of baseline physical test results and to determine the association between physical response after exercise therapy and clinical improvement over 24 weeks.

**Study Design:**

Case-control study; Level of evidence, 3.

**Methods:**

This study recruited 76 consecutive athletes with patellar tendinopathy who were randomized to 2 different programs of exercise therapy for 24 weeks. Athletes underwent a range of physical tests before and during exercise therapy (12 and 24 weeks), including isometric muscle strength (quadriceps and hip abductors), muscle flexibility (quadriceps, hamstrings, soleus, and gastrocnemius), vertical jump height, and visual analog scale (VAS) scores by palpation, after 3 jump trials, and after single-leg squat (VAS-SLS). The Victorian Institute of Sports Assessment–Patella (VISA-P) questionnaire was used as the primary patient-reported outcome. Linear mixed-effect models were used to assess the prognostic value of baseline physical tests. The change in VISA-P score was further dichotomized into clinical responsiveness (≥14 points) and nonresponsiveness (<14 points). Multiple linear and logistic regression models were performed to evaluate associations between physical response and clinical improvement.

**Results:**

Of the 76 included patients, 67 (88%) had complete follow-ups. The estimated mean VISA-P score increased by 23 points (95% CI, 19-28 points) after 24 weeks. No association was found between any baseline physical test results and a 24-week change in VISA-P score (all *P*_interaction_ > .2, using the likelihood ratio test). Improvement in VAS-SLS after exercise therapy was not associated with VISA-P improvement after adjustment (β = −1.76; *P* = .01; Bonferroni-corrected *P* = .10; *R*^2^ = 36.3%). No associations were found between changes in other physical test results and clinical improvement (all *P* > .05).

**Conclusion:**

In patients with patellar tendinopathy, physical test results including strength and flexibility in the lower limb, jump performance, and pain levels during pain-provoking tests were not identified as prognostic factors for patient-reported outcomes after exercise therapy. Similarly, changes in physical test results were not associated with changes in patient-reported outcomes after adjustments. These results do not support using physical test results to estimate prognosis or monitor treatment response.

**Registration:**

NCT02938143 (ClinicalTrials.gov identifier).

Patellar tendinopathy (PT) has a prevalence of 17% in the general population,^3^ and in sports involving jumping, the prevalence rises to 45% in volleyball players and 32% in basketball players.^17^ It has a considerable effect on work productivity (impaired in 58% of people with PT) and sports performance (55% of athletes with PT have to reduce or stop their preferred sports activity).^11^ The diagnosis of PT is established when focal pain at the inferior of the patella is found, especially during tendon-loading activities.^19,25^ Exercise therapy programs have been recognized as the most effective approach in nonoperative treatment for PT.^18,25,38^

Physical tests can be used for the functional assessment of PT. These tests, such as muscle strength, flexibility, jump performance, and pain scale during provocation activities, can measure various physical characteristics.^10,36^ A range of abnormal physical test characteristics was found to be a risk factor for the onset of PT.^24,36,37^ These physical tests are also frequently used in the clinical setting to provide prognosis or to monitor symptoms and function over time in patients with PT. There are, however, currently no studies that evaluate whether physical tests can be used to estimate the course of symptoms (prognosis). Information about changes in the results of physical tests during exercise therapy and its association with the course of symptoms is also lacking. Therefore, the primary aim of this study was to determine the prognostic value of the physical test results measured before starting exercise therapy (baseline) on the change in patient-reported symptoms (difference between baseline and 24 weeks’ follow-up). The secondary aim was to assess the association between the change in physical test results and the change in patient-reported symptoms over 24 weeks.

## Methods

### Study Design and Setting

The study was designed (ClinicalTrials.gov; NCT02938143) and conducted at the Erasmus Medical Center, Rotterdam, the Netherlands. The study was approved by the ethics committee of the Erasmus MC University Medical Center, and all patients provided written informed consent before participation. Patients were enrolled from January 2017 to July 2019. For this specific study, we adhered to the Strengthening the Reporting of Observational studies in Epidemiology guidelines for reporting cohort studies and to the minimum reporting standards for tendinopathy studies according to the International Consensus (ICON) Statement for Tendinopathy.^31^ This study is a secondary analysis using data from the JUMPER study, a large clinical randomized controlled trial designed to investigate the effect of progressive tendon-loading exercises (PTLEs) and eccentric exercise therapy (EET) in athletes with PT for 24 weeks.^6^ The PLTE program consisted of 4 stages: isometric, isotonic, energy storage, and sport-specific exercises. Patients undergoing the EET program served as a control group, and this program consisted of 2 stages: a pain-provoking single-leg squat on a decline board with a 25° slope and sport-specific exercises. When patients completed the sport-specific exercises, they were advised to perform maintenance exercises during the return-to-sports phase. Both exercise programs had a minimum duration of 24 weeks. Additionally, patients were asked to perform exercises targeting risk factors for PT in both study arms. The common treatment principles for tendinopathy involving education and load management advice were given in both groups. Details about the exercise programs, education, and load management advice can be found in the supplementary files of a previously published work.^6^

### Participants

Consecutive patients aged 18 to 35 years with the diagnosis of PT were enrolled. The main inclusion criteria were a clinical diagnosis of PT (pain on loading and palpation pain on the inferior part of the patella), which had to be confirmed by ultrasound findings (tendon structural changes or anterior-posterior thickness >6 mm in grayscale ultrasound and or increased vascularity by power Doppler), and patients with <80 of 100 points on the Victorian Institute of Sports Assessment–Patella (VISA-P) questionnaire. The main exclusion criteria were other coexisting knee pathologies and a history of joint injection therapy in the preceding 12 months. Further details about the eligibility criteria are provided in Appendix 1 (available in the online version of this article).

### Patient-Reported Outcome

The primary outcome was to assess symptom severity change in both groups by a patient-reported outcome measure using a translated and validated Dutch VISA-P questionnaire, ranging from 0 to 100 points. A higher score represents fewer symptoms. The minimal clinically important difference (MCID) for the change of VISA-P score among athletes with PT is 14 points or higher.^12^ The VISA-P questionnaire was completed by patients at baseline and 12 and 24 weeks of follow-up.

### Assessment of Physical Tests

**Figure 1.**
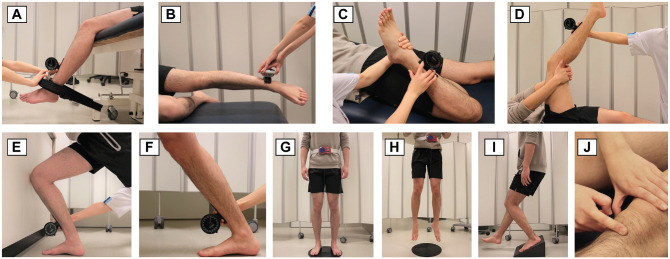
(A) Maximal isometric voluntary contraction (MVC) of the quadriceps muscles was measured with the participant in a seated position on the examination table with both legs hanging over the edge of the table. A fixation band and a plurimeter were used to perform the measurements. Participants were asked to have a straight back and to put both hands on their shoulders. Once the knee flexion angle was at 60° (measured by a plurimeter), the fixation belt was fixed at this position. Participants were asked to put pressure on the dynamometer by contracting their quadriceps while the examiner stabilized the dynamometer. (B) MVC of the hip abductor muscles was measured with the participant in a side-lying position, with the contralateral leg at 90° of flexion. The participant was asked to support his or her head with one hand, and the other hand was used to grasp the edge of the examination table. The participants was asked to put pressure on the dynamometer by contracting the hip abductor muscles against the resistance of the examiner. (C) Quadriceps flexibility was conducted in a prone position. The examiner identified the maximum passive knee flexion angle. Anterior pelvic tilt motion was prevented. (D) Hamstring flexibility was performed in a supine position. The maximum active knee extension angle was dictated by the patient, with a fully extended contralateral leg. (E and F) Participants were asked to lunge in front of the wall. The maximum ankle dorsiflexion range of motion was measured. Soleus flexibility was measured when participants were asked to lunge the knee and touch the wall as far as possible without lifting the heel. The gastrocnemius flexibility was measured by straightening the knee with the ankle at the maximum dorsiflexion angle, still without lifting the heel. (G and H) Participants were asked to wear an adjusted belt connected by a piece of rope before performing 3 trials of the vertical jump. Then, they were asked to land on the mat and report their visual analog scale (VAS) score for maximum pain afterward. The height (cm) was shown on the screen of the device. (I) The single-leg squat was conducted on a 25° decline board. Participants were asked to keep an upright trunk and squat up to 90° of knee flexion. Their VAS score for maximum pain was reported afterward. (J) Tenderness by palpation was assessed using patellar tilting and palpation with the other hand.

Physical test outcomes were collected successively on the same day after the patients completed the VISA-P questionnaire. The coordinating investigator (S.J.B.) assessed the physical test results at baseline and 12 and 24 weeks. A description of the specific physical tests is provided below and shown in Figure 1.

#### Lower Limb Muscle Strength

The maximal isometric voluntary contraction (MVC) was used to assess the strength of the quadriceps and hip abductor muscles by means of a handheld dynamometer (MicroFet 2; Hoggan Health Industries).^30^ The isometric contraction was held for 3 seconds and delivered against the resistance by the examiner (S.J.B.). The highest score over 2 MVC trials was recorded (N) and normalized by body weight (kg) for analysis. The strength of the quadriceps muscles was assessed with the patients seated and the knee at 60° of flexion (Figure 1A). In addition, hip abductor muscle strength was measured in the side-lying position (Figure 1B). Both tests have been shown to have good intraobserver reliability (intraclass correlation coefficient [ICC], 0.73-0.98).^5,28^

#### Lower Limb Flexibility

The flexibility of the lower limb was measured using a plurimeter (Dr Jules Rippstein). The flexibility of the quadriceps muscles was assessed with patients in a prone position by measuring the maximum passive knee flexion angle^33^ (Figure 1C). The reliability of this test in patients with PT is still unknown. The active knee extension test was used to measure the flexibility of the hamstring muscles with patients in a supine position (Figure 1D). This test has been reported to have good intraobserver reliability (ICC, 0.84) in patients with PT.^10^ The flexibility of the soleus muscle (or ankle dorsiflexion angle) was measured by assessing the angle of the tibial shaft during a weightbearing dorsiflexion lunge test (Figure 1E). This test has been shown to have excellent intraobserver reliability (ICC, 0.98).^10^ The flexibility of the gastrocnemius (Figure 1F) was evaluated by calculating the maximum ankle dorsiflexion in the weightbearing technique with the knee extended. This test has been shown to have good intraobserver reliability (ICC, 0.77-0.89) and high interobserver reliability (ICC, 0.95).^27^

#### Performance Test

The maximal jump height was used as a performance test (Figure 1, G and H). We did not require a specific technique. The patients had to jump as high as possible in a vertical direction using both legs (a countermovement before the jump was allowed). The maximal jump height of 3 trials^23^ was recorded in centimeters using a digital vertical jump meter (Takei 5406 Jump-MD; Takei Scientific Instruments Co). The intraobserver reliability of vertical jumps ranged from 0.93 to 0.98.^23^

#### Pain Provocation Tests

A visual analog scale (VAS) score of 0 to 10 was used to quantify pain levels during specific tests (Figure 1, H-J). Pain provocation tests included VAS on palpation (VAS-palpation), after 3 jump trials (VAS-3-jumps), and after single-leg squat (VAS-SLS).^29^ The order of these tests was always the same, with VAS-palpation being the first pain provocation test and VAS-SLS being the last pain provocation test. The maximal VAS score during or directly after these tests was recorded. The reliability of these tests in PT is unknown.

### Statistical Analysis

The first author (J.D.) was conducting statistical analyses and was not involved in performing the physical tests or collecting the VISA-P scores. Statistical analyses under intention to treat were performed. Normality tests were performed by visual check using histograms or normal Q-Q plots and also assessed by the Shapiro-Wilk test. Estimated means and standard deviations were reported for continuous variables with normal distribution. Otherwise, medians and interquartile ranges were used. We performed a 2-sample *t* test and Mann-Whitney *U* test to determine between-group differences at baseline as appropriate.

We used a linear mixed-effect model to evaluate the change in clinical symptoms (VISA-P score) and physical test results in both groups over 24 weeks. The fixed-effect included time, study arms, baseline clinical characteristics (symptom duration and sports activity [Cincinnati Sports Activity Scale]),^4^ sex, body mass index, and age. Individual participant level and time (constant up to 24 weeks) were included as random effects. In addition to these basic models, each baseline physical test result was added to investigate its association with the estimated change in VISA-P score. Interaction between each of these physical test results and time was tested and retained in the model only if the *P* value was significant (<.05) by the likelihood ratio test. To visualize these associations for illustration purposes, we used an arbitrary split using the median value in each of the baseline physical test results. No sensitivity analysis was performed, as the linear mixed-effect model can provide an unbiased result under the assumption that missing data occurred at random.

Linear regression models were used to assess the association between the change in physical test results and the change in VISA-P score over 24 weeks. In addition, we further dichotomized the change in VISA-P score into clinical responsiveness (≥14 points) and nonresponsiveness (<14 points) by MCID, and logistic regression analyses were further used to assess whether these physical changes were associated with the occurrence of clinical responsiveness. All models were adjusted with the aforementioned baseline clinical predictors and additional baseline VISA-P score. In case of missing data in VISA-P scores or physical test results >5%, prediction linear mixed-effect models were used to impute missing values (Appendix 1, available online). We also performed a sensitivity analysis based on a complete case data set in which missing data were excluded.

Residuals were tested to investigate the assumptions of models. Collinearity and outliers were also tested for the linear regression model. In case of multiple testing, a Bonferroni correction method was used.^13^*P* values <.05 were considered statistically significant. All analyses were done using R software Version 2022.4.2.1 (The R Foundation for Statistical Computing). Packages used included “nlme,”“glm,”“emmeans,”“car,”“ggplot2,” and “psych.”

## Results

### Patient Characteristics

**Figure 2.**
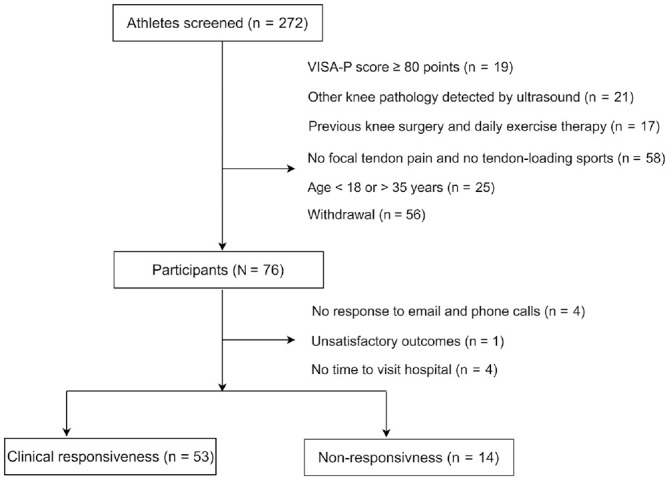
Flow diagram. VISA-P, Victorian Institute of Sports Assessment–Patella.

A total of 76 athletes were included. A summary of baseline characteristics is shown in Table 1, using a recommended report format in tendinopathy research.^31^ Nine athletes (12%) were lost to follow-up at 24 weeks (Figure 2). For baseline physical tests, there were missing values only for VAS-palpation (n = 11 missing; 14%). In addition, baseline physical test results and VISA-P score between clinical responsiveness and nonresponsiveness after 24 weeks are also presented in Appendix 2 (available online). No imbalances were found in physical test results at baseline.

**Table 1:** Baseline Characteristics of 76 Athletes^*a*^

	Value
Demographics	
Age, y, mean (SD)	25 (4)
Male sex	58 (76)
Weight, kg, mean (SD)	81.8 (12.3)
Height, cm, mean (SD)	184.7 (9.3)
BMI, mean (SD)	23.9 (2.9)
Tendinopathy descriptors	
Bilateral tendon involvement	32 (42)
Symptom duration, wk, median [IQR]	104 [49-208]
VISA-P score, mean (SD)	55 (13)
CSAS sports activity level^*b*^	
Level 1: 4-7 d/wk	17 (22)
Level 2: 1-3 d/wk	59 (78)
Imaging for assisting diagnosis	
Use of ultrasound and MRI	76 (100)
Medication use	
Use of painkillers	11 (15)
General health	
EQ VAS, median [IQR]^*c*^	85 [75-90]

a Data are presented as n (%) unless otherwise indicated. BMI, body mass index; CSAS, Cincinnati Sports Activity Scale; EQ, Euro Qol; MRI, magnetic resonance imaging; VAS, visual analog scale; VISA-P, Victorian Institute of Sports Assessment–Patella.

b According to Barber-Westin et al.^4^

c The EQ VAS is part of the EQ-5D and measures a patient's perception of one's overall current health on a vertical VAS (0-100 points), with a higher score representing better general health status.

### Prognostic Value of Baseline Physical Results

**Figure 3.**
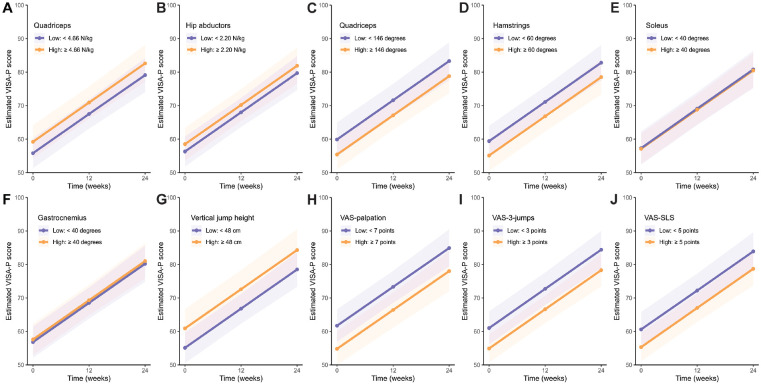
(A-J) Visualization of the prognostic value of baseline physical test results. The prognostic value of each baseline physical test result on the progression of the Victorian Institute of Sports Assessment–Patella (VISA-P) score (0-100 points) over 24 weeks is depicted by dichotomizing baseline physical test results into low and high levels using the median value for illustration purposes. Plots represent the progression of VISA-P scores over 24 weeks by different levels of baseline physical test results. Dots represent marginal estimated means by mixed-effect model without time × physical test interaction. Shaded areas represent the 95% CIs after the Bonferroni correction. There was not a significant difference in the rate of increase in VISA-P between low and high baseline physical test results. VAS, visual analog scale; VAS-palpation, VAS by palpation test; VAS-SLS, VAS after single-leg squat test; VAS-3-jumps, VAS after 3 jump trials.

There was a significant increase in VISA-P score in the whole group, with an increase of 23 points (95% CI, 19-28 points; Bonferroni-corrected *P* < .001) from baseline to 24 weeks (Appendix 3, available online). To evaluate whether any baseline physical test result was associated with the increase in VISA-P score over 24 weeks, we tested interactions of time with each of the physical results in each multivariable linear mixed-effect model (Table 2). The results showed that these baseline physical test results were not significant prognostic factors of VISA-P improvement (all *P*_interaction_ > .2), despite lower VAS-3-jumps scores being significantly associated with higher VISA-P scores at 12 or 24 weeks (β = −2.05; 95% CI, −3.06 to −1.04; Bonferroni-corrected *P* < .01). These results were also visualized by dichotomizing baseline physical test results into low and high levels using the median value for illustration purposes, indicating that the rate of increase in VISA-P score throughout the 24 weeks was constant in all subgroups according to the baseline physical test result levels (Figure 3 and Appendix 4, available online).

**Table 2:** Prognostic Mixed-Effect Model of Baseline Physical Test Results for VISA-P Improvement^*a*^

Test	Time × Physical Test Results^*b*^		Physical Test Results^*c*^	
	*P* Value^*d*^	LRT	β (95% CI)	*P* Value^*d*^
Quadriceps strength, N/kg	.258	1.28	1.39 (–1.33 to 4.12)	.322
Hip abductor strength, N/kg	.327	0.96	1.00 (–5.42 to 7.42)	.761
Quadriceps flexibility, deg	.323	0.98	−0.18 (–0.59 to 0.23)	.393
Hamstring flexibility, deg	.852	0.03	−0.10 (–0.36 to 0.16)	.461
Soleus flexibility, deg	.417	0.66	0.10 (–0.30 to 0.50)	.623
Gastrocnemius flexibility, deg	.395	0.72	−0.19 (–0.57 to 0.18)	.321
Vertical jump height, cm	.675	0.18	0.12 (–0.22 to 0.46)	.505
VAS-palpation	.834	0.04	−0.38 (–1.48 to 0.72)	.507
VAS-3-jumps	.750	0.10	−2.05 (−3.06 to −1.04)	<.001
VAS-SLS	.867	0.03	−1.64 (−2.81 to −0.47)	.008

a LRT, likelihood ratio statistic; VAS, visual analog scale; VAS-palpation, VAS by palpation test; VAS-SLS, VAS after single-leg squat test; VAS-3-jumps, VAS after 3 jump trials; VISA-P, Victorian Institute of Sports Assessment–Patella.

b Interaction terms (time × each physical test result) were tested using the likelihood ratio test, representing the effect of the physical test results on the VISA-P score over time.

c The effect of the physical test results from models without the time × physical test interaction.

d These *P* values were not adjusted by multiple tests. The Bonferroni-corrected *P* value is used, where the raw *P* values are multiplied by the number of tests (n = 10). *P* values <.05 were considered statistically significant.

### Association Between the Changes in Physical Test Results and the Change in VISA-P Score

The longitudinal changes in each physical test result are summarized in Appendix 3 (available online). The aforementioned physical tests, including muscle strength, flexibility, and pain provocation results, improved significantly over time (all Bonferroni-corrected *P* < .001), while no statistically significant longitudinal change in jump height was found (β = 1; 95% CI, –1 to 2; Bonferroni-corrected*P* = .836).

**Figure 4.**
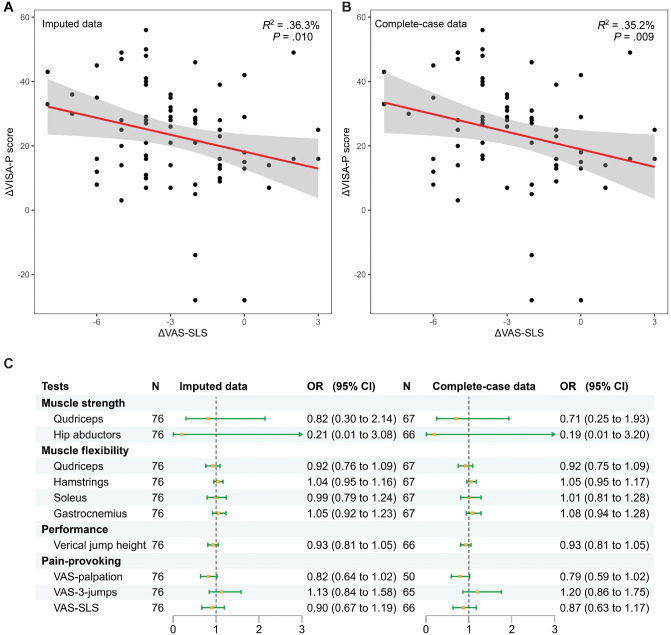
The association between change in physical test results and clinical improvement. (A and B) The associations between the change in the Victorian Institute of Sports Assessment–Patella (ΔVISA-P) score and the change in the visual analog scale single-leg squat test (ΔVAS-SLS) score were evaluated by linear regression models using imputed and complete-case data, adjusted with study arms, sex, body mass index, age, and previous symptom duration, sports activity, and baseline VISA-P scores. Lines represent regression lines. Shaded areas represent the 95% CIs. The *P* values are not adjusted by Bonferroni correction. (C) Forest plots of multivariable logistic regression to make an association between the change in physical test results and the occurrence of clinical responsiveness (change of VISA-P score ≥14 points) using imputed and complete-case data sets. OR, odds ratio; VAS-palpation, VAS by palpation test; VAS-3-jumps, VAS after 3 jump trials.

In the multivariable linear regression models, only a decreased VAS-SLS score was significantly associated with an increase in VISA-P score (β = −1.76; 95% CI, −3.09 to −0.43; *P* = .01; *R*^2^ = 36.3%) (Figure 4A). However, this association did not reach statistical significance after Bonferroni correction (*P* = .1). No associations were found between other physical test result changes and VISA-P improvement (Appendix 5, available online). Furthermore, 58 athletes (76%) were categorized as clinically responsive based on the VISA-P score criterion (change of VISA-P score ≥14 points after 24 weeks), and 18 athletes (24%) as nonresponsive. We ran supplementary analyses on the association between the change in physical test results and the occurrence of clinical responsiveness. In the multivariable logistic regression models, no significant associations were found between the change in any physical test result and clinical responsiveness (all *P* > .05) (Figure 4). Sensitivity analyses performed by complete-case data confirmed the robustness of our outcomes (Appendix 5, available online).

## Discussion

This is the first large-scale study unraveling the association between physical test results and change in a patient-reported outcome in athletes with PT. We found that none of the baseline physical test results were identified as a prognostic factor on clinical outcome after exercise treatment. Specifically, baseline differences in lower limb muscle strength, flexibility, jump performance, and pain level during pain-provoking tests were not associated with an improvement in VISA-P score over 24 weeks. In addition, for athletes receiving exercise therapy over time, we observed a significant but small increase in isometric muscle strength of quadriceps and hip abductors, lower limb flexibility, and decreased pain level during provocation tests. Furthermore, a decreased VAS-SLS was associated with an increase in VISA-P, while there was a lack of statistical significance after multiple testing corrections. Other physical test changes after exercise treatment were not associated with the change in patient-reported outcomes after 24 weeks.

These findings are essential because prognostic factors for predicting clinical outcomes after exercise treatment for PT are lacking. In previous studies, some physical test results have been identified as risk factors for developing PT. These include weakness in quadriceps and hip abductor strength; reduced quadriceps, hamstrings, and ankle flexibility; and greater vertical jump performance.^8,24,36,37^ While these data could be valuable in informing studies on prognostic factors, it should be stressed that risk factors and prognostic factors are different entities. In the clinical setting, pain provocation tests, such as palpation or single-leg squat, are widely used to aid in confirming a diagnosis. It is therefore relevant to investigate whether these easy-to-perform tests could aid in providing a prognosis or monitoring the condition. To date, exercise treatment has been suggested as the first-line therapy for symptomatic PT.^21,25^ However, subgroups of patients will not benefit from exercise therapy.^15,18^ Detecting possible prognostic factors could help clinicians to not only estimate the prognosis but also develop the decision rule to select subgroups of patients with certain characteristics who are most likely to benefit from exercise treatment for PT.^14^

### Prognostic Value of Baseline Physical Test Results

To our knowledge, this is the first study to evaluate whether pretreatment physical test results can predict a 24-week change in VISA-P score for athletes with PT performing exercise therapy. A previous cohort study had identified a set of demographic predictors of 14-week VISA-P change after exercise treatment for PT.^38^ The prognosis was worse for participants with older age, a longer duration of symptoms, and higher training volume after eccentric exercise. In our study, after adjusting these predefined demographic predictors and exercise types, we found no association between these pretreatment physical tests and a 24-week change in VISA-P score. Furthermore, we did not detect any difference in baseline physical test results between athletes who achieved clinical responsiveness (minimal change in VISA-P score ≥14 points) and those who did not (minimal change in VISA-P score <14 points). Collectively, these findings suggest that these pretreatment physical test results may not influence the course of clinical response to exercise treatment. A possible explanation for this finding is that, rather than physical features, other stronger prognostic factors (such as psychological factors) might be crucial components for treatment effectiveness in tendinopathy.^20^ Because exercise therapy can induce pain, it might result in fear and prevent the achievement of meaningful clinical changes in some patients.^16,25^ One cross-sectional study reported that psychological outcomes such as pain catastrophizing and kinesiophobia had a negative influence on the VISA-P score.^34^ Thus, it is reasonable to assume that even if initial physical impairments may affect clinical outcomes, their effect is probably counteracted by these more essential prognostic factors.

### Association Between Physical Response and Clinical Improvement

Clinical improvement after exercise therapy has been shown in both short-term and long-term follow-ups.^1,6,32^ However, whether this clinical improvement is a result of the improvement in physical properties is still unclear. The VISA-P score is the primary or most commonly used outcome to quantify treatment response in patients with PT,^7^ incorporating pain, function, and ability to play sports. Knowing this relationship will provide information on whether these physical tests could be used to monitor the effectiveness of exercise therapy.

#### Lower Limb Muscle Strength and Flexibility

We observed a constant but small increase in lower limb muscle strength and flexibility after exercise therapy over 24 weeks. Several studies have also reported a physical change only with a shorter follow-up duration (12 weeks).^1,32,35^ Agergaard et al^1^ found an increased quadriceps strength after exercise therapy for PT, contrary to Sprague et al^35^ and Ruffino et al,^32^ who reported no change in this muscle strength. In addition, only 1 study^32^ reported muscle flexibility (ankle dorsiflexion) after exercise therapy, and no change was identified. These discrepancies in muscle response may be due to the different exercise programs used in the abovementioned studies. However, we found that these physical improvements were not associated with VISA-P increase or meaningful clinical change. A potential explanation for the lack of association may be the small magnitude of change value observed in physical tests, indicating that more sensitive and advanced equipment may be needed to detect changes in muscle strength response during exercise treatment.

#### Performance Test

As for vertical jump height, despite the fact that Sprague et al^35^ reported a jump height improvement after 12 weeks, our data showed no change in vertical jump height, similar to other previous studies.^1,32^ It is interesting to note that jump performance in our study population did not improve given that muscle strength and flexibility were both increased after exercise therapy; that may be because other factors, such as arm swing or trunk angle, can also affect vertical jump height.^22^ Also, psychological readiness might affect the performance of a maximum jump in athletes recovering from PT.^2^

#### Pain Provocation Tests

In line with a previous study,^1^ we also found a reduced VAS score during pain-provoking tests over 24 weeks in our study population. However, only the decline in VAS-SLS was associated with the change in VISA-P score over 24 weeks. As most of the VISA-P items comprise pain scores, it may be reasonable to assume that the VAS-SLS decline may predict clinical improvement. However, it is important to note that this result was not statistically significant after Bonferroni correction, in the setting of a relatively moderate strength of association between these 2 outcomes (*R*^2^ = 36.3%). One possible explanation is that while pain reduction is a critical goal of treatment for PT, it may not necessarily be related to a reduction in the underlying tendon pathology, which is more directly reflected by the VISA-P score.^9^ Furthermore, none of the pain reduction during these tests was associated with the probability of achieving meaningful clinical changes. Taken together, our outcomes suggest that pain provocation test results may not be useful for solely monitoring treatment response.

### Strengths and Limitations

The major strength of our study is that this is a large prospective study performed in athletes with PT, with structured data collection by a single trained researcher (S.J.B.). The VISA-P score was administered in a standardized way before performing physical tests. Thus, a potential influence on physical test results was avoided as much as possible. Most of the physical test methods have good reliability, and measurement was conducted by the same person, which can reduce the measurement error. Additionally, we performed a sensitivity analysis based on the imputation of data and complete-case data, which enhanced the robustness of our results.

The limitations of our study should also be addressed. First, although a prospective study is the preferable design to answer prognostic questions, a randomized trial can be implemented by combining intervention and control groups and then adjusting the treatment variable in the prognostic model.^26^ However, the generalizability may be reduced when these results are used in athletes whose characteristics are not similar to those of the enrolled participants, because strict inclusion criteria are used in a trial. Second, in our study, other exercise regimens such as isolated heavy slow resistance and isometric exercises^18^ were not included. We strongly believe this does not limit the main findings because there is no strong evidence that one exercise therapy is more beneficial than others.^6,21,25^ Third, we adjusted *P* values using the Bonferroni correction in multiple testing, which may be too conservative to reject true associations.

## Conclusion

In this large prospective cohort study, we identified that none of the baseline physical test results were associated with the change in patient-reported outcome over 24 weeks in athletes with PT after exercise treatment. Although muscle strength, flexibility, and pain level were improved during exercise treatment, no association was found between the change in these physical test results and symptom improvement. Only an improvement in the VAS-SLS test was associated with an improvement in the change in patient-reported outcome, although this association was not statistically significant after adjustment. These results do not support using physical test results to estimate prognosis or monitor treatment response. These findings aid physicians in making an adequate interpretation of the value of physical test results during the recovery of athletes with PT.

## Supplemental Material

sj-pdf-1-ajs-10.1177_03635465231200241 – Supplemental material for Association Between Physical Tests and Patients-Reported Outcomes in Athletes Performing Exercise Therapy for Patellar Tendinopathy: A Secondary Analysis of the JUMPER Study Click here for additional data file. Supplemental material, sj-pdf-1-ajs-10.1177_03635465231200241 for Association Between Physical Tests and Patients-Reported Outcomes in Athletes Performing Exercise Therapy for Patellar Tendinopathy: A Secondary Analysis of the JUMPER Study by Jie Deng, Stephan J Breda, Denise Eygendaal, Edwin HG Oei and Robert-Jan de Vos in The American Journal of Sports Medicine

## References

[1] Agergaard AS Svensson RB Malmgaard-Clausen NM , et al. Clinical outcomes, structure, and function improve with both heavy and moderate loads in the treatment of patellar tendinopathy: a randomized clinical trial. Am J Sports Med. 2021;49(4):982-993. 33616456 10.1177/0363546520988741

[2] Aizawa J Hirohata K Ohji S Ohmi T Koga H Yagishita K . Factors associated with psychological readiness to return to sports with cutting, pivoting, and jump-landings after primary ACL reconstruction. Orthop J Sports Med. 2020;8(11):2325967120964484. 10.1177/2325967120964484 PMC7678401 33244476

[3] Albers IS Zwerver J Diercks RL Dekker JH Van den Akker-Scheek I . Incidence and prevalence of lower extremity tendinopathy in a Dutch general practice population: a cross sectional study. BMC Musculoskelet Disord. 2016;17:16. 26759254 10.1186/s12891-016-0885-2 PMC4711046

[4] Barber-Westin SD Noyes FR . Assessment of sports participation levels following knee injuries. Sports Med. 1999;28(1):1-10. 10461708 10.2165/00007256-199928010-00001

[5] Boling MC Padua DA Marshall SW Guskiewicz K Pyne S Beutler A . A prospective investigation of biomechanical risk factors for patellofemoral pain syndrome: the Joint Undertaking to Monitor and Prevent ACL Injury (JUMP-ACL) cohort. Am J Sports Med. 2009;37(11):2108-2116. 19797162 10.1177/0363546509337934 PMC2860575

[6] Breda SJ Oei EHG Zwerver J , et al. Effectiveness of progressive tendon-loading exercise therapy in patients with patellar tendinopathy: a randomised clinical trial. Br J Sports Med. 2021;55(9):501-509. 33219115 10.1136/bjsports-2020-103403 PMC8070614

[7] Chen PC Wu KT Chou WY , et al. Comparative effectiveness of different nonsurgical treatments for patellar tendinopathy: a systematic review and network meta-analysis. Arthroscopy. 2019;35(11):3117-3131.e2. 10.1016/j.arthro.2019.06.017 31699265

[8] Cook JL Kiss ZS Khan KM Purdam CR Webster KE . Anthropometry, physical performance, and ultrasound patellar tendon abnormality in elite junior basketball players: a cross-sectional study. Br J Sports Med. 2004;38(2):206-209. 15039260 10.1136/bjsm.2003.004747 PMC1724788

[9] Cook JL Rio E Purdam CR Docking SI . Revisiting the continuum model of tendon pathology: what is its merit in clinical practice and research? Br J Sports Med. 2016;50(19):1187-1191. 27127294 10.1136/bjsports-2015-095422 PMC5118437

[10] Crossley KM Thancanamootoo K Metcalf BR Cook JL Purdam CR Warden SJ . Clinical features of patellar tendinopathy and their implications for rehabilitation. J Orthop Res. 2007;25(9):1164-1175. 17469196 10.1002/jor.20415

[11] De Vries AJ Koolhaas W Zwerver J , et al. The impact of patellar tendinopathy on sports and work performance in active athletes. Res Sports Med. 2017;25(3):253-265. 28391723 10.1080/15438627.2017.1314292

[12] Hernandez-Sanchez S Hidalgo MD Gomez A . Responsiveness of the VISA-P scale for patellar tendinopathy in athletes. Br J Sports Med. 2014;48(6):453-457. 23012320 10.1136/bjsports-2012-091163

[13] Jafari M Ansari-Pour N . Why, when and how to adjust your P values? Cell J. 2019;20(4):604-607. 30124010 10.22074/cellj.2019.5992 PMC6099145

[14] Kent P Cancelliere C Boyle E Cassidy JD Kongsted A . A conceptual framework for prognostic research. BMC Med Res Methodol. 2020;20(1):172. 32600262 10.1186/s12874-020-01050-7 PMC7325141

[15] Kettunen JA Kvist M Alanen E Kujala UM . Long-term prognosis for jumper's knee in male athletes: a prospective follow-up study. Am J Sports Med. 2002;30(5):689-692. 12239003 10.1177/03635465020300051001

[16] Kvist J Silbernagel KG . Fear of movement and reinjury in sports medicine: relevance for rehabilitation and return to sport. Phys Ther. 2022;102(2):PZAB272. 10.1093/ptj/pzab272 34971375

[17] Lian OB Engebretsen L Bahr R . Prevalence of jumper's knee among elite athletes from different sports: a cross-sectional study. Am J Sports Med. 2005;33(4):561-567. 15722279 10.1177/0363546504270454

[18] Lim HY Wong SH . Effects of isometric, eccentric, or heavy slow resistance exercises on pain and function in individuals with patellar tendinopathy: a systematic review. Physiother Res Int. 2018;23(4):e1721. 10.1002/pri.1721 29972281

[19] Malliaras P Cook J Purdam C Rio E . Patellar tendinopathy: clinical diagnosis, load management, and advice for challenging case presentations. J Orthop Sports Phys Ther. 2015;45(11):887-898. 26390269 10.2519/jospt.2015.5987

[20] Mallows A Debenham J Walker T Littlewood C . Association of psychological variables and outcome in tendinopathy: a systematic review. Br J Sports Med. 2017;51(9):743-748. 27852585 10.1136/bjsports-2016-096154

[21] Marigi EM Buckley P Razi F , et al. Patellar tendinopathy: critical analysis review of current nonoperative treatments. JBJS Rev. 2022;10(3):e21.00168. doi:10.2106/JBJS.RVW.21.00168 35358114

[22] Markovic G . Does plyometric training improve vertical jump height? A meta-analytical review. Br J Sports Med. 2007;41(6):349-355. 17347316 10.1136/bjsm.2007.035113 PMC2465309

[23] Markovic G Dizdar D Jukic I Cardinale M . Reliability and factorial validity of squat and countermovement jump tests. J Strength Cond Res. 2004;18(3):551-555. 15320660 10.1519/1533-4287(2004)18<551:RAFVOS>2.0.CO;2

[24] Mendonça LD Ocarino JM Bittencourt NFN Macedo LG Fonseca ST . Association of hip and foot factors with patellar tendinopathy (jumper’s knee) in athletes. J Orthop Sports Phys Ther. 2018;48(9):676-684. 29792104 10.2519/jospt.2018.7426

[25] Millar NL Silbernagel KG Thorborg K , et al. Tendinopathy. Nat Rev Dis Primers. 2021;7(1):1. 33414454 10.1038/s41572-020-00234-1

[26] Moons KGM Royston P Vergouwe Y Grobbee DE Altman DG . Prognosis and prognostic research: what, why, and how? BMJ. 2009;338:B375. 10.1136/bmj.b375 19237405

[27] Munteanu SE Strawhorn AB Landorf KB Bird AR Murley GS . A weightbearing technique for the measurement of ankle joint dorsiflexion with the knee extended is reliable. J Sci Med Sport. 2009;12(1):54-59. 17888733 10.1016/j.jsams.2007.06.009

[28] Piva SR Fitzgerald K Irrgang JJ , et al. Reliability of measures of impairments associated with patellofemoral pain syndrome. BMC Musculoskelet Disord. 2006;7:33. 16579850 10.1186/1471-2474-7-33 PMC1557500

[29] Purdam CR Cook JL Hopper DM Khan KM ; VIS Tendon Study Group. Discriminative ability of functional loading tests for adolescent jumper's knee. Phys Ther Sport. 2003;4(1):3-9.

[30] Rathleff MS Samani A Olesen JL Roos EM Rasmussen S Madeleine P . Effect of exercise therapy on neuromuscular activity and knee strength in female adolescents with patellofemoral pain—an ancillary analysis of a cluster randomized trial. Clin Biomech (Bristol, Avon). 2016;34:22-29. 27054583 10.1016/j.clinbiomech.2016.03.002

[31] Rio EK Mc Auliffe S Kuipers I , et al. ICON PART-T 2019-International Scientific Tendinopathy Symposium Consensus: recommended standards for reporting participant characteristics in tendinopathy research (PART-T). Br J Sports Med. 2020;54(11):627-630. 31519545 10.1136/bjsports-2019-100957

[32] Ruffino D Malliaras P Marchegiani S Campana V . Inertial flywheel vs heavy slow resistance training among athletes with patellar tendinopathy: a randomised trial. Phys Ther Sport. 2021;52:30-37. 34384941 10.1016/j.ptsp.2021.08.002

[33] Sanchis-Alfonso V Coloma-Saiz J Herrero-Herrero M Prades-Piñón J Ramírez-Fuentes C . Evaluation of anterior knee pain patient: clinical and radiological assessment including psychological factors. Ann Jt. 2018;3(3):26. doi:10.21037/aoj.2018.03.15

[34] Slagers AJ van Veen E Zwerver J Geertzen JHB Reininga IHF van den Akker-Scheek I . Psychological factors during rehabilitation of patients with Achilles or patellar tendinopathy: a cross-sectional study. Phys Ther Sport. 2021;50:145-152. 34015607 10.1016/j.ptsp.2021.04.010

[35] Sprague AL Couppé C Pohlig RT Snyder-Mackler L Silbernagel KG . Pain-guided activity modification during treatment for patellar tendinopathy: a feasibility and pilot randomized clinical trial. Pilot Feasbility Stud. 2021;7(1):58. 10.1186/s40814-021-00792-5 PMC7905015 33632313

[36] Sprague AL Smith AH Knox P Pohlig RT Grävare Silbernagel K . Modifiable risk factors for patellar tendinopathy in athletes: a systematic review and meta-analysis. Br J Sports Med. 2018;52(24):1575-1585. 30054341 10.1136/bjsports-2017-099000 PMC6269217

[37] van der Worp H van Ark M Roerink S Pepping GJ van den Akker-Scheek I Zwerver J . Risk factors for patellar tendinopathy: a systematic review of the literature. Br J Sports Med. 2011;45(5):446-452. 21367808 10.1136/bjsm.2011.084079

[38] van Rijn D van den Akker-Scheek I Steunebrink M Diercks RL Zwerver J van der Worp H . Comparison of the effect of 5 different treatment options for managing patellar tendinopathy: a secondary analysis. Clin J Sport Med. 2019;29(3):181-187. 31033610 10.1097/JSM.0000000000000520

