# Supplementary material for: Association Between Physical Tests and Patients-Reported Outcomes in Athletes Performing Exercise Therapy for Patellar Tendinopathy: A Secondary Analysis of the JUMPER Study
Source: Am J Sports Med. 2023 Oct 10;51(13):3523–32. doi: 10.1177/03635465231200241 (PMC10623612; doi:10.1177/03635465231200241)
Supplement: sj-pdf-1-ajs-10.1177_03635465231200241 – Supplemental material for Association Between Physical Tests and Patients-Reported Outcomes in Athletes Performing Exercise Therapy for Patellar Tendinopathy: A Secondary Analysis of the JUMPER Study [file sj-pdf-1-ajs-10.1177_03635465231200241.pdf]

Results of Physical Tests Have No Prognostic Value for Patient-reported Outcomes in Athletes Performing Exercise Therapy for Patellar Tendinopathy: A secondary analysis of JUMPER study

Appendix 1

Participants

The study was announced by advertising through local sports organizations, medical and paramedical conferences, websites, newsletters, and emails to professional medical organizations. An online screening was initially used to assess the potential eligibility of applicants, including the Victorian Institute of Sports Assessment for Patellar Tendon (VISA-P) and a self-reported pain map to localize the pain in the region of the anterior knee. A final screening in XX University Medical Center to confirm eligibility for participants presumed eligible included physical examination and grey-scale ultrasound (GSUS) and or power Doppler (PDUS) scanning. Critical inclusion criteria were (1) clinical diagnosis of patellar tendinopathy (localized pain on palpation at the inferior patellar pole and during a single-leg squat, (2) age 18 to 35 years, (3) VISA-P score < 80 out of 100 points, (4) the presence of tendon structural changes and increased anterior-posterior (AP) thickness (diameter >6 mm) by GSUS and or the presence of blood vessels within or around the tendon by PDUS. Exclusion criteria included the following: coexisting other knee or patellar pathology; acute knee injuries; history of knee surgery without full recovery; tendon rupture; location injection; medication (limited to antibiotics for patellar tendon); inability to perform exercise program. Provocation tests were also performed to exclude patellofemoral pain, and patients with bilateral symptoms were also included in this study. Patients with the diagnosis of PT were randomly assigned to PTLE or EET group in a 1:1 ratio.

Imputation by linear mixed-effect model

We used a predicted linear mixed-effect model (LMM) to impute missing data in VISA-P score and physical test results to calculate the change of each variable from 24 weeks to baseline. Each LMM consisted of random effect (individual participant level as random intercept and time as random slope) and fixed effect (time, study arm, sex, BMI, age, prior symptom duration, and sports activity). To impute individual value in each randomization group, we also tested the interaction term (study arm * time) in each LMM, and it was only retained if it was significant at the 5% level after comparing it to the original model by performing the maximum likelihood (ML). The significant P value is 0.05. Residuals were tested to investigate the assumptions of models.

Appendix 2

VISA-P score and physical test results at baseline according to clinical responsiveness

Tests at baseline	Clinical responsiveness (n=53)<br><br>Mean (SD) or median [IQR]	Non-responsiveness (n=14)<br><br>Mean (SD) or median [IQR]	*P* Value
VISA-P score	54 (13)	60 (12)	.093[a]
Muscle strength tests, N/kg			
Quadriceps	4.7 (1.0)	4.5 (1.1)	.543[a]
Hip abductors	2.2 (0.4)	2.2 (0.5)	.808[a]
Muscle flexibility tests, degrees			
Quadriceps	147 (7)	143 (6)	.075[a]
Hamstrings	61 (11)	62 (9)	.767[a]
Soleus	41 (7)	43 (5)	.198[a]
Gastrocnemius	39 (7)	41 (7)	.227[a]
Performance test, cm			
Vertical jump height	50 (9)	48 (11)	.615[a]
Pain provocation tests			
VAS-palpation	6 [4 – 8]	7 [5 – 8]	.875[b]
Missing, n(%)	8	3	
VAS-3-jumps	3 [1 – 5]	3 [2 – 6]	.262[b]
VAS-SLS	5 [3 – 7]	5 [3 - 6]	1.000[b]

VISA-P, Victorian Institute of Sports Assessment for patellar tendons (0-100 point); N/kg, Newton per kilogram; cm, centimeters; VAS, Visual Analog Scale (rating scale [0-10]); VAS-palpation, VAS by palpation test; VAS-3-jumps, VAS after 3 three jump trials; VAS-SLS, VAS after single leg squat test.
[a]Independent t-test
[b]Mann-Whitney U test

Appendix 3

Longitudinal change in physical tests results and VISA-P score in athletes following exercise therapy over 24 weeks

In the following table and figure, the longitudinal changes were calculated using linear mixed-effect model. The fixed effect included time, study arms, baseline clinical characteristics (symptom duration, sports activity [Cincinnati Sports Activity Scale (CSAS)], sex, BMI, and age. Individual participant level and time (constant up to 24 weeks) were included as a random effect.

Estimated VISA-P score and physical test results over 24 weeks[a]

Tests	Baseline		12 weeks		24 weeks		Change[b]	
	n	Estimated mean (95% CI)	n	Estimated mean (95% CI)	n	Estimated mean (95% CI)	Estimated difference (95% CI)	*P* value[b]
VISA-P score	76	57 (53 to 61)	64	69 (65 to 73)	67	81 (76 to 85)	23 (19 to 28)	<.001
Quadriceps strength, N/kg	76	4.6 (4.3 to 4.9)	65	4.9 (4.7 to 5.1)	67	5.2 (5.0 to 5.4)	0.6 (0.4 to 0.8)	<.001
Hip abductors strength, N/kg	76	2.1 (2.0 to 2.3)	65	2.2 (2.1 to 2.3)	66	2.3 (2.2 to 2.4)	0.1 (0.1 to 0.2)	<.001
Quadriceps flexibility, degrees	76	147 (145 to 149)	65	148 (146 to 150)	67	149 (147 to 151)	2 (0.8 to 3.4)	<.001
Hamstrings flexibility, degrees	76	63 (60 to 67)	65	65 (62 to 68)	67	67 (63 to 70)	3 (1 to 6)	<.001
Soleus flexibility, degrees	76	41 (39 to 43)	64	42 (40 to 44)	67	43 (40 to 45)	2 (1 to 3)	<.001
Gastrocnemius flexibility, degrees	76	39 (36 to 41)	64	40 (38 to 42)	67	41 (38 to 43)	2 (1 to 4)	<.001

Vertical jump height, cm	76	47 (45 to 49)	64	47 (45 to 50)	66	48 (45 to 50)	1 (-1 to 2)	.836
VAS-palpation	63	6 (5 to 7)	60	4 (4 to 5)	58	3 (2 to 3)	-3 (-5 to -2)	<.001
VAS-3-jumps	76	(2 to 3)	64	2 (1 to 2)	66	1 (0 to 1)	-2 (-3 to -1)	<.001
VAS-SLS	76	5 (4 to 6)	65	4 (3 to 4)	66	2 (2 to 3)	-3 (-4 to -2)	<.001

VISA-P, Victorian Institute of Sports Assessment for patellar tendons (0-100 point); N/kg, Newton per kilogram; VAS, Visual Analog Scale (rating scale [0-10]); VAS-palpation, VAS by palpation test; VAS-3-jumps, VAS after 3 three jump trials; VAS-SLS, VAS after single leg squat test; n, number of participants.

[a]Shown is marginal estimated mean from the mixed model.

[b]The change are calculated from value at baseline to those at 24 weeks.

[b]The p-value is Bonferroni-corrected.

Visualization of longitudinal change in physical tests over 24 weeks

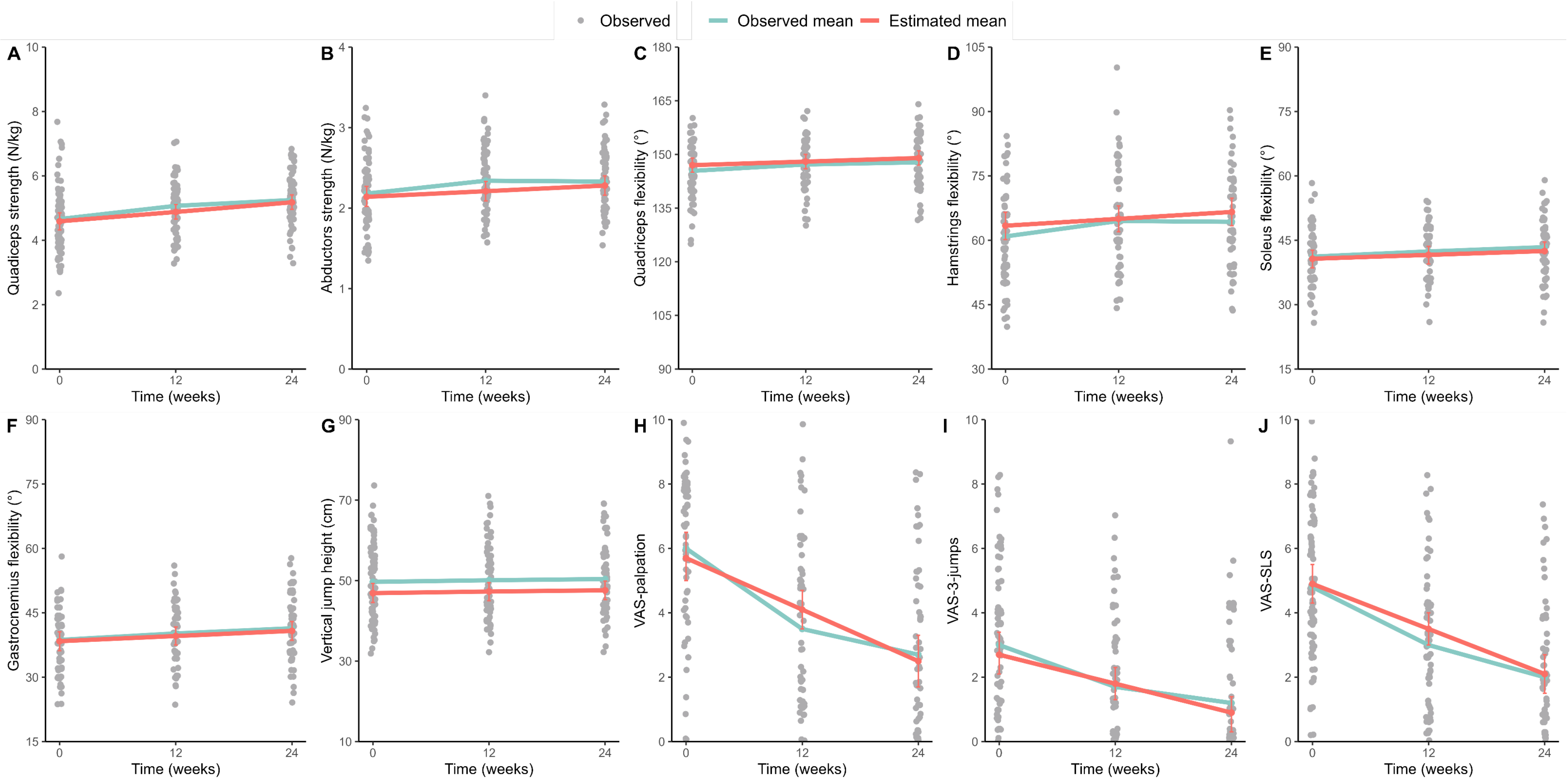

°: Degrees

Observed, raw data; Observed mean, the raw mean; Estimated mean, marginal mean from linear mixed-effect model; VAS-palpation, VAS by palpation test; VAS-3-jumps, VAS after 3 three jump trials; VAS-SLS, VAS after single leg squat test.

Appendix 4

Estimated change in VISA-P score over 24 weeks according to physical test level

For illustration purpose, we also run linear mixed-effect models to report the estimated change in VISA-P score according to baseline physical test level. The fixed effect included time, study arms, baseline clinical characteristics (symptom duration, sports activity [Cincinnati Sports Activity Scale (CSAS)], sex, BMI, and age. Individual participant level and time (constant up to 24 weeks) were included as a random effect. The marginal estimated mean is from linear mixed-effect model without time-by-physical tests interaction.

Tests	VISA-P score at baseline (n=76)	VISA-P score 24 weeks (N=67)	Increase in VISA-P score From baseline 24 weeks	
	Estimated mean (95% CI)	Estimated mean (95% CI)	Estimated change (95% CI)	*P* value[a]
**Quadriceps strength**				
Low < 4.66 N/kg	56 (51 to 60)	79 (74 to 84)	23 (18 to 28)	<0.001
High ≥ 4.66 N/kg	59 (54 to 64)	83 (77 to 88)	23 (18 to 28)	<0.001
**Hip abductors strength**				
Low < 2.20 N/kg	56 (52 to 61)	80 (75 to 85)	23 (19 to 28)	<0.001
High ≥ 2.20 N/kg	59 (54 to 63)	82 (77 to 87)	23 (19 to 28)	<0.001
**Quadriceps flexibility**				
Low < 146 degrees	60 (55 to 65)	83 (78 to 89)	23 (19 to 28)	<0.001
High ≥ 146 degrees	55 (51 to 60)	79 (74 to 84)	23 (19 to 28)	<0.001
**Hamstrings flexibility**				
Low < 60 degrees	59 (55 to 64)	83 (78 to 88)	23 (19 to 28)	<0.001
High ≥ 60 degrees	55 (51 to 60)	79 (73 to 84)	23 (19 to 28)	<0.001
**Soleus flexibility**				
Low < 40 degrees	57 (52 to 62)	81 (75 to 86)	23 (19 to 28)	<0.001

High ≥ 40 degrees	57 (53 to 62)	81 (75 to 86)	23 (19 to 28)	<0.001
**Gastrocnemius flexibility**				
Low < 40 degrees	56 (52 to 61)	80 (75 to 85)	23 (19 to 28)	<0.001
High ≥ 40 degrees	59 (54 to 63)	82 (77 to 87)	23 (19 to 28)	<0.001
**Vertical jump height**				
Low < 48 cm	55 (51 to 60)	79 (73 to 84)	23 (19 to 28)	<0.001
High ≥ 48 cm	61 (55 to 67)	84 (78 to 91)	23 (19 to 28)	<0.001
**VAS-Palpation**				
Low < 7 points	62 (57 to 67)	85 (79 to 91)	23 (18 to 28)	<0.001
High ≥ 7 points	55 (50 to 60)	78 (72 to 84)	23 (18 to 28)	<0.001
**VAS-3-jumps**				
Low < 3 points	61 (56 to 66)	84 (79 to 90)	23 (19 to 28)	<0.001
High ≥ 3 points	55 (51 to 59)	78 (73 to 83)	23 (19 to 28)	<0.001
**VAS-SLS**				
Low < 5 points	61 (55 to 66)	84 (78 to 90)	23 (18 to 28)	<0.001
High ≥ 5 points	55 (51 to 60)	79 (74 to 84)	23 (18 to 28)	<0.001

VISA-P, Victorian Institute of Sports Assessment for patellar tendons (0-100 point); N/kg, Newton per kilogram; cm, centimeters; VAS, Visual Analog Scale (rating scale [0-10]); VAS-palpation, VAS by palpation test; VAS-3-jumps, VAS after 3 three jump trials; VAS-SLS, VAS after single leg squat test.

[a]The p-value is Bonferroni-corrected.

Appendix 5

Associations between the change in physical test results and the change in VISA-P score or clinical responsiveness

In the following tables, we present multiple linear regression and logistic regression analyses to investigate associations. To confirm the robustness of our outcomes, we present sensitivity analyses for both models, using imputed data and complete-case data.

The association between the change in physical test results and the change in VISA-P score using imputed data

ΔVariables	Δ VISA-P[a]			Clinical Responsiveness[b]	
	β (95% CI)	*P* value[c]	R^2 (%)	OR (95% CI)	*P* value[c]
Quadriceps muscle strength, N/kg	-1.62 (-6.19 to 2.95)	.482	30.3	0.82 (0.30 to 2.14)	.683
Hip abductors muscle strength, N/kg	-13.49 (-27.21 to 0.22)	.054	33.5	0.21 (0.01 to 3.08)	.267
Quadriceps flexibility, degrees	-0.43 (-1.23 to 0.37)	.285	30.9	0.92 (0.76 to 1.09)	.382
Hamstrings flexibility, degrees	0.04 (-0.39 to 0.47)	.858	29.8	1.04 (0.95 to 1.16)	.401
Soleus flexibility, degrees	-0.93 (-2.05 to 0.47)	.101	32.5	0.99 (0.79 to 1.24)	.903
Gastrocnemius flexibility, degrees	-0.13 (-0.76 to 0.51)	.695	29.9	1.05 (0.92 to 1.23)	.505
Vertical jump height, cm	-0.21 (-0.83 to 0.42)	.517	30.2	0.93 (0.81 to 1.05)	.242
VAS-palpation	-0.63 (-1.59 to 0.33)	.195	31.5	0.82 (0.64 to 1.02)	.083
VAS-3-jumps	-1.07 (-2.60 to 0.46)	.167	13.8	1.13 (0.84 to 1.58)	.424
VAS-SLS	-1.76 (-3.09 to -0.43)	.010	36.3	0.90 (0.67 to 1.19)	.468

VISA-P, Victorian Institute of Sports Assessment for patellar tendons (0-100 point); VAS, Visual Analog Scale (rating scale [0-10]); VAS-palpation, VAS by palpation test; VAS-3-jumps, VAS after 3 three jump trials; VAS-SLS, VAS after single leg squat test; N/kg, Newton per kilogram; cm, centimeters; R^2, adjusted R-squared by linear regression models; Δ, the change from baseline to 24 weeks; OR, odd ratio.
[a]Using linear regression models, adjusted with study arms, sex, gender, BMI, symptom duration, sports activity, and baseline VISA-P score.
[b]Using logistic regression models. Clinical responsiveness is defined as the minimal clinically meaningful change based on the VISA-P score (the minimal change of VISA-P ≥ 14 points). We dichotomized VISA-P into clinical responsiveness (≥ 14 points) and non-responsiveness (< 14 points). Models are adjusted with sex, gender, BMI, symptom duration, sports activity, and baseline VISA-P score.
[c]These p values were not adjusted by multiple testing.The Bonferroni corrected p values is using multiplying the raw p values by the number of the tests (n=10). The significant p-value is 0.05.

Sensitivity analyses for association between the change in physical test results and the change in VISA-P score using multiple linear regression models

ΔVariables	ΔVISA-P score					
	Imputed data			Complete-case data		
	n	β (95% CI)	*P* value[a]	n	β (95% CI)	*P* value[a]
Quadriceps muscle strength, N/kg	76	-1.62 (-6.19 to 2.95)	.482	67	-1.73 (-6.73 to 3.27)	.492
Hip abductors muscle strength, N/kg	76	-13.49 (-27.21 to 0.22)	.054	66	-14.41 (-29.48 to 0.66)	.061
Quadriceps flexibility, degrees	76	-0.43 (-1.23 to 0.37)	.285	67	-0.46 (-1.31 to 0.40)	.293
Hamstrings flexibility, degrees	76	0.04 (-0.39 to 0.47)	.858	67	0.03 (-0.44 to 0.51)	.884
Soleus flexibility, degrees	76	-0.93 (-2.05 to 0.47)	.101	67	-0.88 (-2.07 to 0.31)	.146
Gastrocnemius flexibility, degrees	76	-0.13 (-0.76 to 0.51)	.695	67	-0.09 (-0.77 to 0.60)	.804
Vertical jump height, cm	76	-0.21 (-0.83 to 0.42)	.517	66	-0.16 (-0.85 to 0.54)	.657
VAS-palpation	76	-0.63 (-1.59 to 0.33)	.195	50	-1.05 (-2.18 to 0.07)	.066
VAS-3-jumps	76	-1.07 (-2.60 to 0.46)	.167	65	-0.97 (-2.71 to 0.77)	.269
VAS-SLS	76	-1.76 (-3.09 to -0.43)	.010	66	-2.03 (-3.52 to -0.53)	.009

VISA-P, Victorian Institute of Sports Assessment for patellar tendons (0-100 point); N/kg, Newton per kilogram; VAS, Visual Analog Scale (rating scale [0-10]); VAS-palpation, VAS by palpation test; VAS-SLS, VAS after single leg squat test, VAS-3-jumps, VAS after 3 three jump trials. N/kg, Newton per kilogram; cm, centimeters; Δ, the change from baseline to 24 weeks; n, number of participants.

[a]These p values were not adjusted by multiple testing.The Bonferroni corrected p values is using multiplying the raw p values by the number of the tests (n=10). The significant p-value is 0.05.

Sensitivity analyses for association between the change in physical test results and clinical responsiveness

ΔVariables	Clinical responsiveness					
	Imputed data			Complete-case data		
	n	OR (95% CI)	*P* value[a]	n	OR (95% CI)	*P* value[a]
Quadriceps muscle strength, N/kg	76	0.82 (0.30 to 2.14)	.683	67	0.71 (0.25 to 1.93)	.506
Hip abductors muscle strength, N/kg	76	0.21 (0.01 to 3.08)	.267	66	0.19 (0.01 to 3.20)	.265
Quadriceps flexibility, degrees	76	0.92 (0.76 to 1.09)	.382	67	0.92 (0.75 to 1.09)	.338
Hamstrings flexibility, degrees	76	1.04 (0.95 to 1.16)	.401	67	1.05 (0.95 to 1.17)	.372
Soleus flexibility, degrees	76	0.99 (0.79 to 1.24)	.903	67	1.01 (0.81 to 1.28)	.931
Gastrocnemius flexibility, degrees	76	1.05 (0.92 to 1.23)	.505	67	1.08 (0.94 to 1.28)	.322
Vertical jump height, cm	76	0.93 (0.81 to 1.05)	.242	66	0.93 (0.81 to 1.05)	.241
VAS-palpation	76	0.82 (0.64 to 1.02)	.083	50	0.79 (0.59 to 1.02)	.084
VAS-3-jumps	76	1.13 (0.84 to 1.58)	.424	65	1.20 (0.86 to 1.75)	.303
VAS-SLS	76	0.90 (0.67 to 1.19)	.468	66	0.87 (0.63 to 1.17)	.358

VISA-P, Victorian Institute of Sports Assessment for patellar tendons (0-100 point); N/kg, Newton per kilogram; VAS, Visual Analog Scale (rating scale [0-10]); VAS-palpation, VAS by palpation test; VAS-SLS, VAS after single leg squat test, VAS-3-jumps, VAS after 3 three jump trials; N/kg, Newton per kilogram; cm, centimeters; Δ, the change from baseline to 24 weeks; OR, odd ratio; n, number of participants.

[a]These p values were not adjusted by multiple testing.The Bonferroni corrected p values is using multiplying the raw p values by the number of the tests (n=10). The significant p-value is 0.05.
